# A virtual reality time reproduction task for rodents

**DOI:** 10.3389/fnbeh.2022.957804

**Published:** 2022-08-10

**Authors:** Josphine Henke, Virginia L. Flanagin, Kay Thurley

**Affiliations:** ^1^Faculty of Biology, Ludwig-Maximilians-Universität München, Munich, Germany; ^2^Bernstein Center for Computational Neuroscience Munich, Munich, Germany; ^3^German Center for Vertigo and Balance Disorders, Ludwig-Maximilians-Universität München, Munich, Germany

**Keywords:** interval timing, time reproduction, magnitude estimation, virtual reality, gerbil, animal behavior

## Abstract

Estimates of the duration of time intervals and other magnitudes exhibit characteristic biases that likely result from error minimization strategies. To investigate such phenomena, magnitude reproduction tasks are used with humans and other primates. However, such behavioral tasks do not exist for rodents, one of the most important animal orders for neuroscience. We, therefore, developed a time reproduction task that can be used with rodents. It involves an animal reproducing the duration of a timed visual stimulus by walking along a corridor. The task was implemented in virtual reality, which allowed us to ensure that the animals were actually estimating time. The hallway did not contain prominent spatial cues and movement could be de-correlated from optic flow, such that the animals could not learn a mapping between stimulus duration and covered distance. We tested the reproduction of durations of several seconds in three different stimulus ranges. The gerbils reproduced the durations with a precision similar to experiments on humans. Their time reproductions also exhibited the characteristic biases of magnitude estimation experiments. These results demonstrate that our behavioral paradigm provides a means to study time reproduction in rodents.

## 1. Introduction

Timing skills include the ability to estimate the duration of time intervals. One method for testing such an ability is *time reproduction*, in which the participant of the experiment is presented with a target interval and then must reproduce its length by some behavioral response (Grondin, 2010). Such a task thus requires the reproduction of the magnitude of the stimulus, here the duration, and therefore is a magnitude estimation experiment. Behavioral responses in magnitude estimation experiments show characteristic psychophysical effects (Petzschner et al., 2015). Most famously they include the *regression effect* also known as regression to the mean, central tendency, or Vierordt's law (von Vierordt, 1868; Hollingworth, 1910). It states that, given a range of stimuli, small stimuli are overestimated while large stimuli are underestimated. For ranges that comprise larger stimulus values, regression becomes more pronounced, called range effect (Teghtsoonian and Teghtsoonian, 1978). As a result, the same stimuli lead to different responses on average when embedded in different stimulus distributions (Jazayeri and Shadlen, 2010; Petzschner and Glasauer, 2011). Finally, also the Weber-Fechner law has consequences on magnitude estimation: errors increase with the size of the stimulus, which leads to what is called *scalar variability* (Weber, 1851; Fechner, 1860).

Over the past decade, the behavioral effects seen in magnitude estimation have been linked to error minimization strategies acting on the fusion of a stimulus estimate with prior knowledge (Jazayeri and Shadlen, 2010; Petzschner and Glasauer, 2011; Cicchini et al., 2012; Shi et al., 2013; Bausenhart et al., 2014; Petzschner et al., 2015; Thurley, 2016), which led to a renewed interest in these phenomena, including neuroscientific studies of their neural substrates (Wiener et al., 2016; Sohn et al., 2019; Henke et al., 2021; Meirhaeghe et al., 2021; Sohn and Narain, 2021). In a recently published study, we investigated the neural basis of time reproduction in rodent medial prefrontal cortex (Henke et al., 2021). In this study, we used a novel time reproduction task that we developed for rodents. Here, we extend this work with more behavioral experiments and show that Mongolian gerbils (*Meriones unguiculatus*) can be trained to reproduce stimuli from different stimulus ranges. We find regression and range effects as well as scalar variability similar to other studies that have used humans and other primates as model animals.

## 2. Materials and methods

### 2.1. Animals

The time reproduction experiments were conducted with seven female adult Mongolian gerbils (*Meriones unguiculatus*) from a wild-type colony at the local animal house (referred to by IDs 8727, 8728, 8729, 8730, 10525, 10526, and 10570). Training started at an age of at least 4 months. The gerbils were housed individually on a 12-h light/dark cycle, and all behavioral training and recording sessions were performed in the light phase of the cycle. The animals received a diet maintaining them at about 85–95% of their free feeding weight. All experiments were approved according to national and European guidelines on animal welfare (Reg. von Oberbayern, District Government of Upper Bavaria; reference number: AZ 55.2-1-54-2532-10-11).

### 2.2. Experimental apparatus

**Figure 1:**
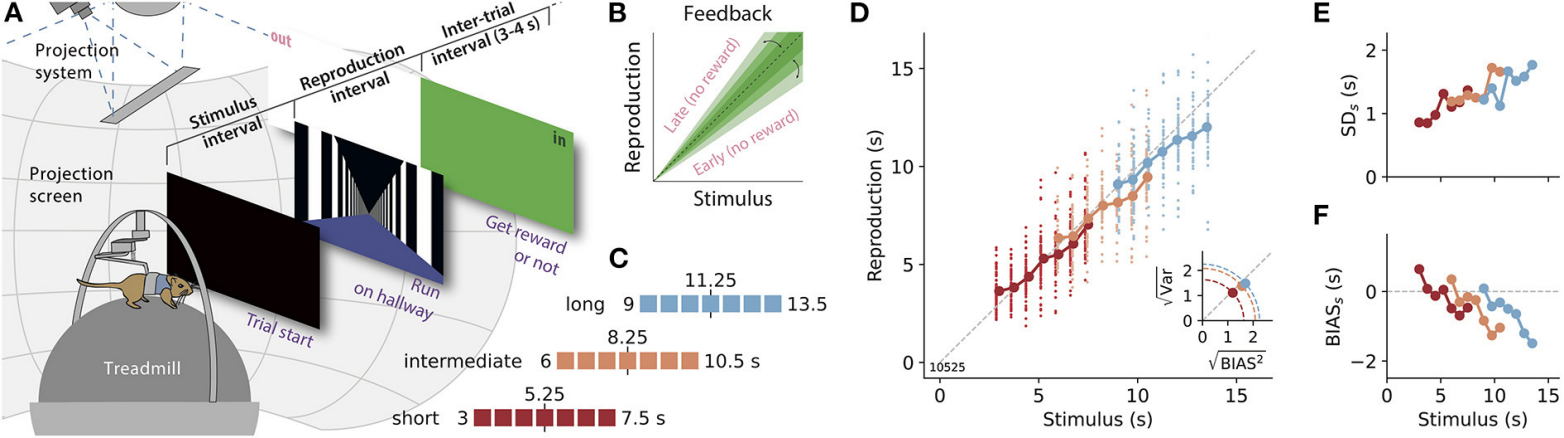
A time reproduction task for rodents. **(A)** Experimental apparatus and task. A gerbil was placed on a treadmill surrounded by a projection screen. Trials started with a timed stimulus (black screen). Then a virtual corridor appeared and the animal had to reproduce the time interval by walking. If the reproduction was close to the stimulus duration (“in”), a food reward was delivered and the entire screen was set to green for 3–4 s before another trial was initiated; otherwise, the screen was set to white (“out”). **(B)** The range for positive feedback, i.e., the error-tolerance window, was narrowed/widened after each in/out response. **(C)** Stimulus durations for one session were randomly sampled from one of three discrete uniform distributions with seven values each. Upper and lower borders are given as numbers. These stimulus ranges differed only by their mean values (marked by a vertical black solid line and a number). Colors identify range and will be used throughout the article. **(D)** Time reproductions of an example gerbil. Individual reproduced values are given as small dots and averages for each stimulus as large dots connected by a solid line. Gray dashed line marks equality. *Inset:* Square roots of the average variance (i.e., the SD) vs those for average squared bias for the three stimulus distributions. The distance from the origin of the coordinate system is the RMSE. Along the dashed quarter circles, RMSE would be constant. **(E)** SD for each stimulus duration (SD_*s*_). **(F)** Average bias, i.e., response − stimulus, for each stimulus (BIAS_*s*_).

Experiments were done with a virtual reality (VR) setup for rodents (Figure 1A). For a detailed description refer to Thurley et al. (2014). In brief, the setup consisted of an air-suspended styrofoam sphere that acted as a treadmill. The rodent was fixated above the sphere with a harness that left head and legs freely movable, and the legs resting on the sphere. When the animal moved its legs, the sphere rotated. These rotations were detected by infrared sensors and fed into a computer to generate and update a visual virtual scene. The scene was displayed *via* a projector onto a screen surrounding the treadmill. We used Vizard Virtual Reality Toolkit (v5, WorldViz, http://www.worldviz.com) for real-time rendering; the virtual environment was designed with Blender (v2.49b, http://www.blender.org/). Animals were rewarded with food pellets (20 mg Purified Rodent Tablet, banana and chocolate flavor, TestDiet, Sandown Scientific, UK) that were automatically delivered and controlled by the VR software.

### 2.3. Behavioral paradigm

We implemented a time reproduction task, in which a rodent had to estimate the duration of a timed stimulus and reproduce it by moving along a virtual corridor. The basic procedure is displayed in Figure 1A: At the beginning of a trial, the projection switched to black for a specific duration. Animals were trained to measure this duration and not to move meanwhile. Afterward, the visual scene changed, a virtual corridor appeared and the animal had to reproduce the measured duration by moving along the corridor. The animal decided freely when to start or stop the reproduction phase. Typically, animals began walking after a few seconds. These “reaction times” correlated only weakly with the stimulus and the reproduced stimulus, and in only a few sessions (Supplementary Figure S2C). Only when the animal continuously moved on the treadmill for at least 1 s, the start of this movement was counted as the beginning of reproduction. To finish reproduction, the animal had to stop for more than 0.5 s. This 0.5 s were not counted to the reproduced duration. These procedures ensured that short movements and stops were not taken as responses. In Supplementary Figure S1, we display movement data from an example session. After the reproduction epoch, the animals were given feedback on their performance (refer to Section 2.3.2). Immediately thereafter, the next trial started automatically.

#### 2.3.1. Stimulus distributions

Stimulus durations were randomly chosen from one of three stimulus ranges in a session. These stimulus distributions were discrete and uniform with seven different durations each (Figure 1C): the “short” range included durations between 3 and 7.5 s (3, 3.75, 4.5, 5.25, 6, 6.75, and 7.5), the “intermediate” range reached from 6 to 10.5 s (6, 6.75, 7.5 8.25, 9, 9.75, and 10.5), and the “long” range contained values from 9 to 13.5 s (9, 9.75, 10.5, 11.25, 12, 12.75, and 13.5). Three stimulus durations thus overlapped between the short and the intermediate and the intermediate and the long range, respectively.

#### 2.3.2. Feedback and reward

At the end of each trial, a gerbil received feedback on its time-reproduction performance. Following the reproduction epoch, the entire projection screen was either set to green (positive, “in”) or white (negative, “out”) for 3–4 s. For an “in” response, the animal was additionally rewarded with a food pellet. To receive such a reward, the reproduction had to be sufficiently close to the stimulus duration, i.e., (1±*k*) × stimulus. The width of this error-tolerance window depended on the stimulus duration (cf. Jazayeri and Shadlen, 2010), to capture that errors increase with duration, i.e., scalar variability. Across an experimental session, tolerance *k* was reduced by −3% when a reward was given and extended by +3% otherwise (Figure 1B). At the beginning of a session, *k* was set to the value from the end of the previous session. As a consequence of the adaptive error-tolerance window, reward rates lay roughly between 50 and 75% (Supplementary Figure S2A) and animals reached average error tolerance windows of 20% or smaller (Supplementary Figure S2B).

#### 2.3.3. Avoidance of spatial solution strategies

The virtual corridor was designed to exclude landmark-based strategies. It was infinite and had a width of 0.5 m. The walls of 0.5 m height were covered with a repetitive pattern of black and white stripes, each with a height to width ratio of 1:5. The floor was homogeneously colored in medium light-blue and the sky was black.

By randomly changing the gain between an animal's movement on the treadmill and movement in VR, movement time was de-correlated from the virtual distance traveled at the same time. This was done to prevent path integration as a strategy for task solving. Gain values were uniformly sampled between 0.25 and 2.25. Distributions of virtual speed and running speed on the treadmill as well as their correlations with stimulus duration, reproduced duration and bias can be found in Supplementary Figures S3A,B. Running speed was (mostly negatively) correlated in less than 25% of the sessions to stimulus and reproduction.

### 2.4. Behavioral training and testing

Naive gerbils were familiarized with the VR setup in the infinite virtual corridor for five to ten sessions (i.e., about 2 weeks, cf. Thurley et al., 2014). These sessions were used to get the animals comfortable wearing the harness, accepting the restraint, using the treadmill, and receiving automatically delivered rewards. The animals were body fixed with the harness such that turning around on the treadmill was prevented. They were trained to walk along the maze in only one direction. The reward apparatus delivered pellets automatically at several positions along the maze to encourage walking. In later sessions, the distances that had to be covered between rewards were increased. The success of the familiarization was evaluated by the experimenter. We started with two groups of four animals each. One animal could not be successfully accustomed to the VR setup and was, therefore, taken out of the experiments, such that we performed the study with seven animals.

Then, we exposed the animals to the timing task with the structured trial. In each trial, the animals saw the timed black screen and only afterward the corridor. They were trained to only walk when the corridor was presented. If they walked during the presentation on the black screen, the experimenter blocked the treadmill such that it could not be moved by the animal. This haptic feedback was efficient in teaching the animals to stop walking. As a first step, we presented only stimuli of 3 and 6 s that were easy for the animals to discriminate. The animals had to learn to either walk for a short or a long duration. Positive feedback was initially given with a tolerance of *k* = 50% and training proceeded until values below 30% were reached for at least three subsequent sessions. This training phase took about 1.5 months (ca. 30 sessions). In the second part of the training, we presented the full stimulus range for a few sessions (~1 week), to introduce the animals to stimuli on a continuous scale. Afterward, the test phase started. All animals performed all stimulus ranges. The initial sessions for all animals were with the short stimulus range. Gerbils 8727 and 8730 then performed the intermediate range sessions, followed by the long range sessions, gerbils 8728 and 8729 performed the long range sessions first followed by the intermediate ones; and gerbils 10525, 10526, and 10570 performed interleaved intermediate and long range sessions.

### 2.5. Analysis of behavioral data

To compare behavioral performance across ranges and animals, we calculated different measures. The strength of the regression effect was assessed by the slope of the linear regression between stimuli *s* and their reproductions *r*. A slope of one would correspond to no regression and smaller slopes to stronger regression. The mean squared error MSE(*r*) = E[(*r*−*s*)^2^] gives the deviation between stimuli *s* and reproductions *r*. It can be split into two contributions


$$\begin{array}{l} \text{MSE}(r)={\text{E}}_{s}\left[{\text{VAR}}_{s}\left(r\right)\right]+{\text{E}}_{s}\left[{\text{BIAS}}_{s}^{2}\left(r\right)\right]\\ \;=\underset{\text{VAR}\left(r\right)}{\underset{︸}{{\text{E}}_{s}\left[{\text{E}}_{r}\left[{\left(r-{\text{E}}_{r}\left[r|s\right]\right)}^{2}|s\right]\right]}}+\underset{{\text{BIAS}}^{2}(r)}{\underset{︸}{{\text{E}}_{s}\left[{\left({\text{E}}_{r}\left[r|s\right]-s\right)}^{2}\right]}}, \end{array}$$

where Var*s*(*r*) is the variance and BIAS*s*^2^(*r*) the squared bias of the responses for stimulus *s*, E*s*[·] and E*r*[·] denote expected values over stimuli *s* or responses *r*, e.g., E*r*[*r*∣*s*] is the average response to a stimulus *s*. Partitioning the MSE as above, we separated the general variability in the responses Var(*r*) from systematic biases BIAS^2^(*r*). Note that we always take the square roots of the above parameters, i.e., standard deviation $\sqrt{\text{Var}}=\text{SD}$, $\sqrt{{\text{BIAS}}^{2}}$ and root-mean-square error RMSE, to provide values in seconds. Since $\sqrt{{\text{BIAS}}^{2}}$ does not contain information about the direction of systematic errors, like general under or overestimation, we quantified it with the BIAS(*r*) = E*s*[BIAS_*s*_(*r*)] = E*s*[E*r*[*r*∣*s*]−*s*]. Variability normalized to the stimulus range is measured by the coefficient of variation, which we calculated as $\text{CV}\left(r\right)=\text{E}s\left[\frac{\text{SD}s\left(r\right)}{\text{E}r\left[r|s\right]}\right]$. Again, E*r*[*r*∣*s*] is the average response to a stimulus *s* and SD*s*(*r*) the corresponding SD. The ratio of both values is averaged over all stimuli, denoted by E*s*[·].

**Figure 2:**
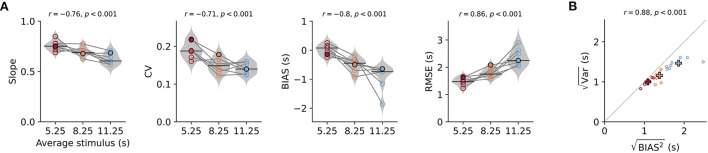
Behavioral characteristics of time reproduction. **(A)** The slope of the linear regression between stimuli and reproductions – quantifying the strength of the regression effect, with values closer to 1 meaning less regression—coefficient of variation (CV), average bias, and RMSE for each animal across stimulus ranges. Values from single animals are displayed as open circles connected by lines. Gray violin plots illustrate distributions over all animals, and black solid lines mark the medians. The color indicates (s)mall, (i)ntermediate, and (l)ong stimulus ranges. The filled markers belong to the data of the animal in Figures 1D–F. Above the panels, the Pearson correlation coefficients of the parameter with the average stimulus duration and corresponding *p*-values are given. **(B)** Correlation between the square roots of the average variance and the average squared bias for the three stimulus ranges across all animals. The distance from the origin of the coordinate system is the RMSE. Crosses mark averages over animals for each range. Color code as in **(A)**. Both values increase with range and are strongly correlated.

**Figure 3:**
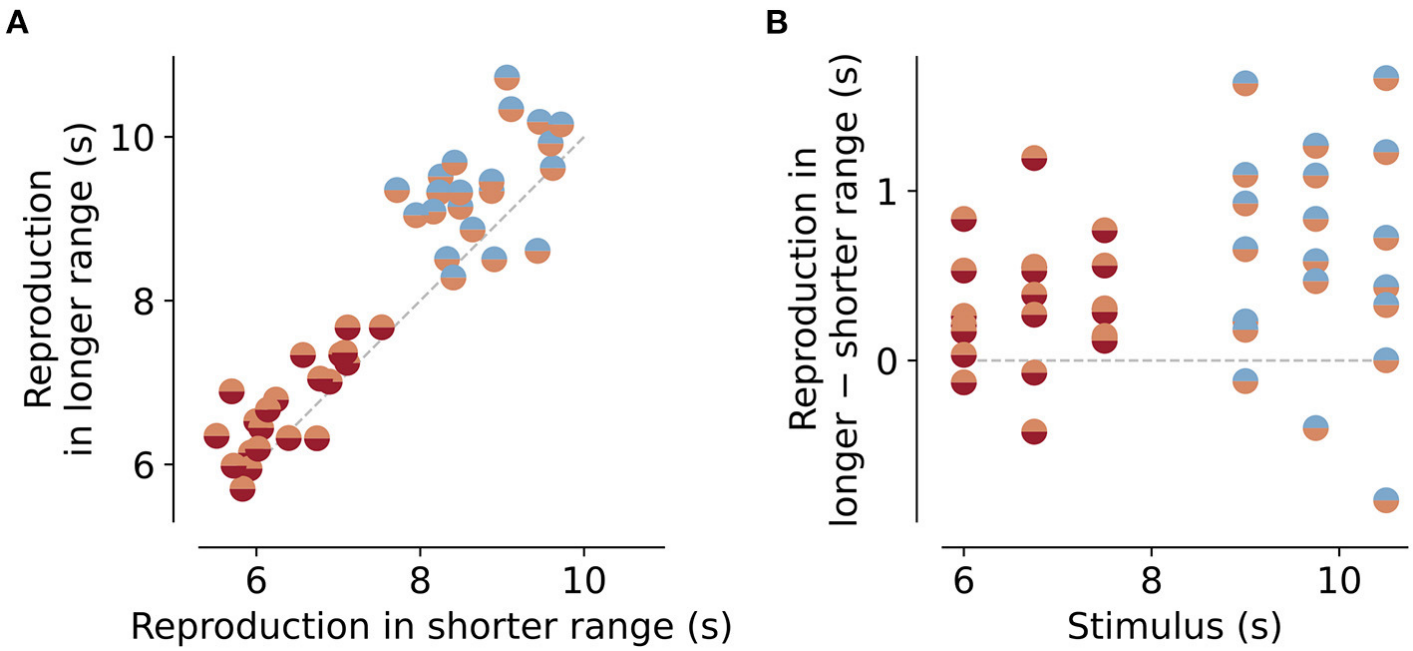
Reproductions for the same stimuli embedded in different stimulus ranges. **(A)** Reproduced values for stimuli that are part of the short and intermediate ranges (orange/red markers) or intermediate and long ranges (blue/orange markers). Data points lie above the equality line (gray dashed line). Therefore, reproductions of the same stimulus are usually bigger when embedded in a longer range than in a shorter one. This is even better visible in the differences between reproductions **(B)**. Gray dashed line marks no difference.

For the pooled data in Figure 2, we first ensured that individual data sets were normally distributed with Shapiro-Wilk tests and tested for equal variances with Levene tests. Then, we performed one-way repeated measures ANOVA. In addition, we calculated Pearson correlation coefficients *r* between the analysis parameter and the average stimulus value for each stimulus range. *Post-hoc* pairwise comparisons were done with Tukey's honestly significant difference (HSD) test. The data in Figure 3 were statistically evaluated with a *t*-test. For all statistical hypothesis tests, a *p*-value of no more than 5% was accepted as significant.

Data analysis was done with Python 3.8 using Matplotlib 3.5, Numpy 1.21, Pandas 1.4, Scipy 1.8, and Statsmodels 0.13.

## 3. Results

We trained gerbils to measure and reproduce the duration of time intervals lasting a few seconds in virtual reality (VR). After the presentation of a timed black screen, the animals had to reproduce its duration by walking along a virtual corridor (Figure 1A). The corridor appeared infinitely long and was covered with black and white stripes to provide the visual impression of movement while preventing landmark-based spatial strategies. To also avoid path integration for task solving, the gain of the coupling between the treadmill and the projection was changed on each trial to de-correlate time and distance traveled. Reproductions were rewarded if they were sufficiently close to the stimulus duration. This error-tolerance window was adjusted after each reproduction. A hit (in) decreased the width of the window and a miss (out) increased it (Figure 1B). The seven gerbils were tested in three different stimulus ranges that only differed by their means (Figure 1C). On average six experimental sessions were conducted in each range, with approximately 50 trials per session.

The gerbils reproduced time intervals close to the stimulus durations, displaying good time reproduction abilities. However, their responses also exhibited typical characteristics of magnitude estimation. In Figures 1D–F, we show example data from one animal. The data of the other animals can be found in Supplementary Figure S4. In Figure 1D, the regression effect is evident in each stimulus range. Moreover, it is amplified in the ranges with longer stimuli, in particular, when one compares the short and the long range. Especially for the long range, stimulus durations were often generally underestimated such that there was no actual overestimation of the shorter stimuli in the range. However, when the width spanned by all reproductions across the entire range is considered it appears compressed compared to the width of the stimulus range. So there is still a regression effect. This results in a slope smaller than one for linear fits between all stimulus durations in a range and their reproduced values. We, therefore, use this slope to quantify the regression effect below. Scalar variability is also consistently present across the three ranges as the SD increases with longer stimulus durations and is independent of the stimulus range (Figure 1E and Supplementary Figure S4).

To describe the effects across animals, we calculated several parameters for each animal and stimulus range. Sessions for the same range were pooled. As mentioned above, we used the slope of linear fits between stimulus durations and their reproduced values to quantify the strength of the regression effect. Values closer to one indicate less regression and values closer to zero mean more regression. The slopes were below one for all animals and ranges, indicating the regression effect, and also displayed the range effect as they decreased with stimulus range (i.e., average stimulus, Figure 2A). We found a significant negative correlation between slope and average stimulus for each range (Pearson's *r* = −0.76, *p* < 0.001) and slopes for the ranges were significantly different from each other [rANOVA *F*_(2, 12)_ = 11.674, *p* = 0.002, Tukey HSD for short vs. intermediate *p* = 0.09, short vs. long *p* < 0.001, intermediate vs. long *p* = 0.0383]. In line with an increased regression effect for longer ranges also the square-root of the mean squared bias $\sqrt{{\text{BIAS}}^{2}}$ increased with stimulus range as well as the RMSE [Figures 2A,B; rANOVA for RMSE *F*_(2, 12)_ = 752, *p* < 0.001 with significant Tukey HSD for all three pairs of ranges].

According to scalar variability, the SD (i.e., the error) of the responses should increase with stimulus magnitude. As already mentioned above, we consistently observed this effect in our animals (Supplementary Figure S4). Similarly, across animals average SD within one range was larger for ranges with longer stimuli and this was correlated with the $\sqrt{{\text{BIAS}}^{2}}$ (Figure 2B). Statistical testing revealed significant differences between ranges [rANOVA *F*_(2, 12)_ = 25.384, *p* < 0.001, Tukey HSD for short vs. intermediate *p* = 0.0866, short vs. long *p* < 0.001, intermediate vs. long *p* < 0.001]. We also normalized variability for the stimulus range by using the coefficient of variation (CV; Figure 2A) and found values comparable to time reproduction performance in humans for millisecond durations (Jazayeri and Shadlen, 2010) and second durations (Thurley and Schild, 2018). There was a mild but significant decrease in CV between the short and the two longer ranges [rANOVA *F*_(2, 12)_ = 15.12, *p* < 0.001, Tukey HSD for short vs. intermediate *p* = 0.002, short vs. long *p* < 0.001] and a corresponding negative correlation (*r* = −0.71, *p* < 0.001).

Time reproductions for our gerbils also displayed general underestimation, which we quantified by the average difference between stimuli and reproductions (BIAS in Figure 2A). The magnitude of this bias became larger (more negative values) for longer stimulus ranges [rANOVA *F*_(2, 12)_ = 11.9234, *p* = 0.0014 Tukey HSD for short vs. intermediate *p* = 0.0161, short vs. long *p* < 0.001, intermediate vs. long *p* = 0.0506].

In the analyses up to now, we pooled the behavioral data for each range across several sessions. A similar picture appeared at the single session level, showing consistent effects across sessions (Supplementary Figure S5). For the animals 10525, 10526, and 10570 variability was larger between sessions than for the other four animals, 8727–8730. Between these two groups, the session order was different. For 8727–8730, experiments for one stimulus range were always performed in blocks before we switched to another range (see Section 2). For animals 10525, 10526, and 10570, the short range sessions were performed first but the intermediate and long ranges were interleaved (semi-randomly chosen by the experimenter), which likely explains the observed variability across sessions.

As a next step, we analyzed the range effect in more detail. The dependence of the regression effect on stimulus range, we found above (“Slope” parameter in Figure 2A), should result in differences in the reproduced values for the same stimuli when embedded in different stimulus ranges. To test this, we compared the mean responses for the stimulus durations that were part of more than one range. For the short and intermediate ranges, these durations were 6, 6.75, and 7.5 s, and for the intermediate and long ranges 9, 9.75, and 10.5 s (Figure 1C). Indeed the same stimulus duration was reproduced as longer when given in an experimental session where durations were drawn from a longer range compared to a shorter one (Figure 3A). The mean of the distribution of these differences was significantly larger than would be expected from a zero-mean normal distribution (Figure 3B). For the short vs. the intermediate range, differences were 0.3±0.3*s* on average [*t*-test, *t*_(20)_ = 4.066, *p* < 0.001], for the intermediate vs. the long range differences were 0.6±0.7*s* on average [*t*-test, *t*_(20)_ = 4.45, *p* < 0.001]. Across session effects can occur, i.e., one range can affect the reproductions in a different range, since the prior or reference may carry over from one session to another. For example, an animal that first receives the short range immediately followed by the long range, may show shorter reproductions in the long range than an animal that did the intermediate range before the long range. To test this we split the data in Figure 3A into three different groups. All animals first performed the sessions of the short range. However, for the next sessions ranges were given in three different ways: (A) intermediate then long, (B) long then intermediate, and (C) randomly interleaving intermediate and long sessions. As Supplementary Figure S6 shows, there was indeed an effect of session order, however, it was surprisingly different from what we expected. Group A showed larger reproductions for the intermediate compared to the short range but shorter reproductions for the long range compared to the intermediate. For group B, the picture was reversed, and in group C with the interleaved ranges, such “range order effects” disappeared. So there is an impact on range order but not in a simple way related to forming the prior. More importantly, if one wants to avoid such effects of range order, they can be mitigated by randomly interleaving sessions of different stimulus ranges. However, since we only had two to three animals in each group, these results should be viewed with some caution.

Finally, we asked whether our gerbils indeed performed time reproduction? Alternatively, they could also count the steps they do on the treadmill for reproduction. They may, thus, have learned to map between stimulus duration and the number of steps to do in the reproduction epoch. Counting steps would correspond to the path length covered by the animals on the treadmill during a trial. To test whether path length could explain our results, we compared the correlations between stimulus and reproduction with that between stimulus and real path length, i.e., the distance covered on the treadmill. In addition, we did the same analysis with virtual path length, i.e., the distance traveled visually in VR. Supplementary Figure S7 displays the results. The correlations of stimulus with either type of path length were always smaller than those with reproduced time. For virtual path length, this demonstrates the efficiency of our de-correlation of movement time from the virtual distance traveled at the same time through the gain changes. For real path length, a few experimental sessions showed similar correlations to those of stimulus duration and reproduced duration but in most sessions the correlations with the real path length were lower, indicating that animals did time reproduction instead of mapping stimulus duration into a movement distance.

## 4. Discussion

We developed a behavioral paradigm to probe time reproduction in rodents. We already used this task in a recently published study in which we investigated the neural basis of time reproduction in the rodent medial prefrontal cortex (Henke et al., 2021). Here, we extended the behavioral experiments and showed that Mongolian gerbils (*Meriones unguiculatus*) can be trained to reproduce stimuli from different stimulus ranges.

The time reproduction task was implemented in a virtual reality system for rodents (Thurley and Ayaz, 2017), which allowed us to use walking on a treadmill as a way for rodents to reproduce intervals. Running, in particular treadmill use, is very attractive even to wild rodents (Meijer and Robbers, 2014). Furthermore, in VR, we could prevent landmark-based and path integration strategies for task solving. The virtual corridor did not contain prominent spatial cues. A technique often used in path integration studies with bees (e.g., Srinivasan et al., 2000) but also gerbils (Kautzky and Thurley, 2016). Moreover, self-motion could be de-correlated from optic flow, so that animals could not learn a mapping between stimulus duration and distance traveled.

To teach our gerbils the timing task, we started training with 3 and 6 s stimuli and then proceeded with the short stimulus range before finally presenting the intermediate and long ranges. The choice of 3 and 6 s and the start with the short range experiments were made for practical reasons. The 3 and 6 s duration are easy to discriminate but they are not lasting too long. For a short range session, more trials can be performed in the same amount of total time as for longer ranges. However, our analyses showed that there may be effects of stimulus range that reach across sessions. In case one wants to avoid such effects, the order in which different ranges are presented could be randomly chosen across several sessions.

Magnitudes of physical stimuli as well as distances and durations are continuous by nature. The ability of humans to estimate a continuum of time intervals is well investigated. In contrast, interval timing in rodents is typically studied with tasks that only test for single durations (*peak procedure*) or compare two durations (Grondin, 2010; Shettleworth, 2010). One famous method is that of *bisection*: two learned standards are used as borders for short and long according to which other stimulus durations have to be categorized. Bisection experiments can be used to determine how precise time intervals can be differentiated but they only provide information about discrimination abilities not about how magnitudes are judged. To our knowledge, our time reproduction task is the first such paradigm for rodents. As we demonstrated, it can be used to investigate the estimation of stimuli on a continuous scale and different stimulus ranges can be tested. Moreover, specific intervals do not need to be trained beforehand. When the gerbils learned the concept of the task, we could provide stimulus durations from a different range without specific training and re-learning. Due to the walking response, the intervals that can be tested have to last several seconds, i.e., time scales that are relevant for behaviors like spatial navigation and related action planning.

The gerbils' behavioral responses exhibited regression and range effects as well as scalar variability similar to studies with humans (Jazayeri and Shadlen, 2010; Petzschner and Glasauer, 2011; Cicchini et al., 2012; Martin et al., 2017; Roach et al., 2017; Thurley and Schild, 2018). Our results thus demonstrate that the typical behavioral effects found in time reproduction and other magnitude estimation experiments are present in gerbils. This is of particular interest if error minimization strategies indeed underly these effects as suggested (Shi et al., 2013; Bausenhart et al., 2014; Petzschner et al., 2015; Thurley, 2016). It would mean that rodents also show error minimisation providing a useful animal model for studying these effects. This could extend the scope beyond pure primate work to comparative studies and demonstrates a ubiquitous presence of error minimization mechanisms across at least the mammalian class.

## Data availability statement

The raw data supporting the conclusions of this article will be made available by the authors, without undue reservation.

## Ethics statement

The animal study was reviewed and approved by Reg. von Oberbayern, District Government of Upper Bavaria; reference number: AZ 50 55.2-1-54-2532-10-11.

## Author contributions

KT and VF envisioned the study and designed the behavioral paradigm. JH performed the experiments. KT and JH analyzed the data. KT wrote the manuscript. All authors contributed to the article and approved the submitted version.

## Funding

This study was supported by BMBF (Federal Ministry of Education and Research, Germany) *via* Bernstein Center Munich (Grant No. 01GQ1004A).

## Conflict of interest

The authors declare that the research was conducted in the absence of any commercial or financial relationships that could be construed as a potential conflict of interest.

## Publisher's note

All claims expressed in this article are solely those of the authors and do not necessarily represent those of their affiliated organizations, or those of the publisher, the editors and the reviewers. Any product that may be evaluated in this article, or claim that may be made by its manufacturer, is not guaranteed or endorsed by the publisher.
