# Supplementary material for: A virtual reality time reproduction task for rodents
Source: Front Behav Neurosci. 2022 Aug 10;16:957804. doi: 10.3389/fnbeh.2022.957804 (PMC9399742; doi:10.3389/fnbeh.2022.957804)
Supplement: Supplementary file 1 [file Data_Sheet_1.pdf]

Supplementary Material

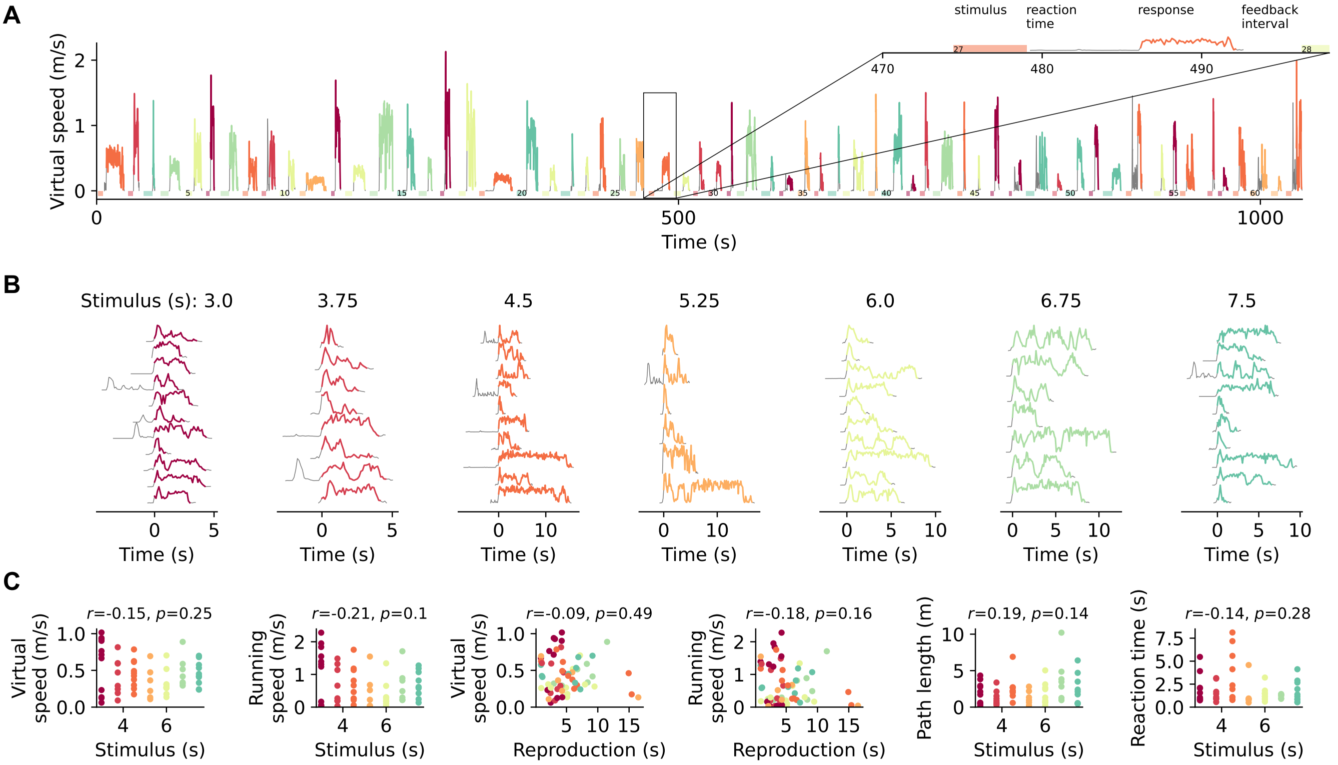

Figure S1. Example session for the short range. (A) Speed of visual movement an animal experiences over the course of a session (virtual speed). Position was tracked during the reproduction epoch but not during measurement (stimulus) and feedback interval. Colored traces mark the intervals included as responses; gray otherwise. Color identifies stimulus. The interval between the end of stimulus presentation and the beginning of the response we call "reaction time". To finish the reproduction the animal had to stop for at least 0.5 s. *Inset:* Magnification of the 27th trial with labelled trial parts. (B) Virtual speed in individual trials sorted by stimulus. (C) Averages for each trial of the virtual speed, the running speed of the animal on the treadmill, the path length covered during reproduction, and the reaction time sorted by stimulus or reproduction. Color identifies stimulus. Pearson's r and p-values are given on top of each panel.

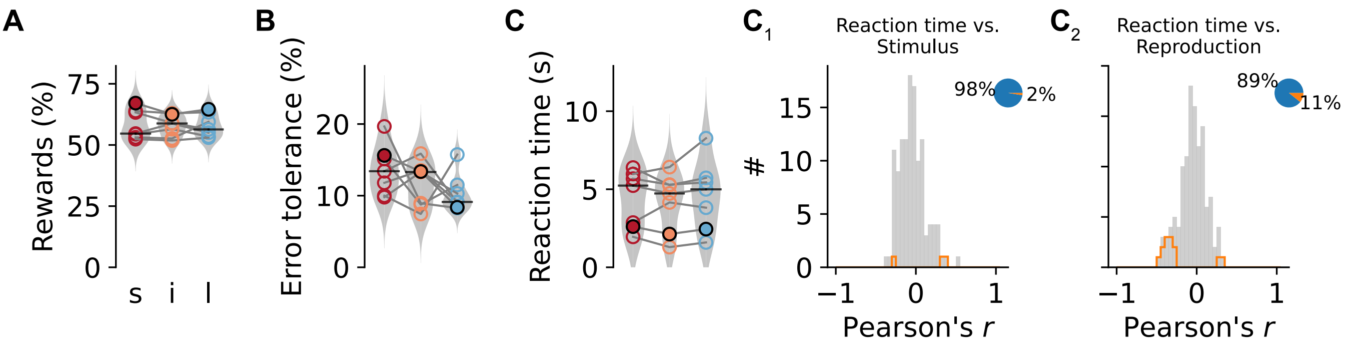

Figure S2. Task specific behavioral characteristics. (A) Percentage of rewards for each animal across stimulus ranges. Markers connected by lines belong to data of one animal. Color identifies (s)mall, (i)ntermediate and (l)ong ranges. Violin plots illustrate the distribution of the population. A solid black line marks the median. The filled markers belong to the data of the example animal displayed in Figure 1D-F in the main text. (B) Average width of the error-tolerance window for each animal across ranges. (C) Average reaction time (start of reproduction after stimulus offset) for each animal across ranges. (C_1&C_2) Distributions of Pearson's correlation coefficients between reaction time and stimulus or reproduced values for each session (pooled across animals and stimulus ranges). Histograms are displayed in gray with significant values delimited by an orange outline ($p \leq 5\%$). Pie plots show significant (orange) and non-significant (blue) fractions.

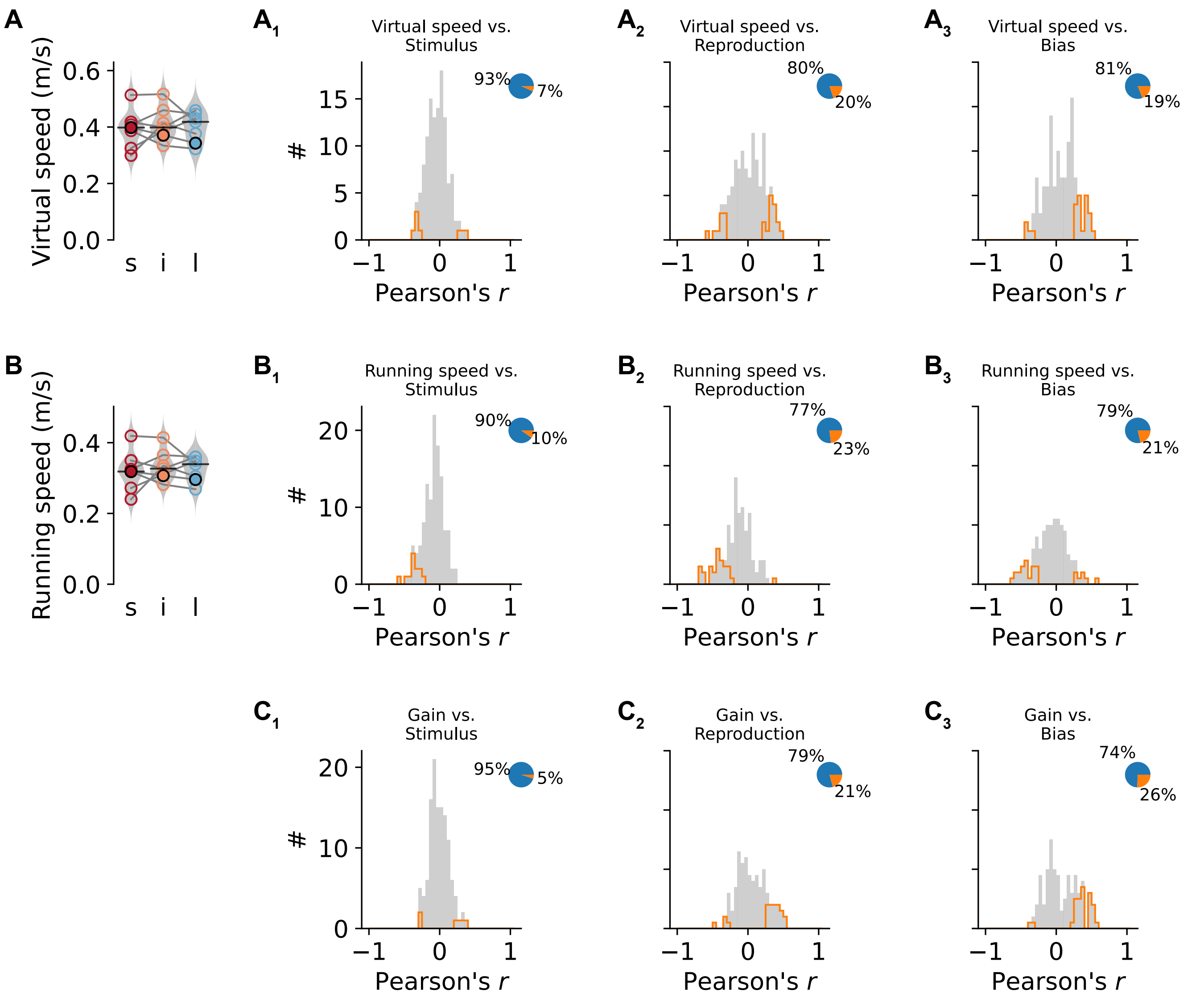

Figure S3. Effects of movement speed. (A) Average virtual speed in each session and distributions of Pearson's correlations with stimulus (A_1), reproduction (A_2), and bias, i.e. reproduction - stimulus duration (A_3). Data displayed as in Fig. S2. (B) Same as (A) for running speed of the animals on the treadmill. (C) Same as (A) for gain between visual projection and treadmill.

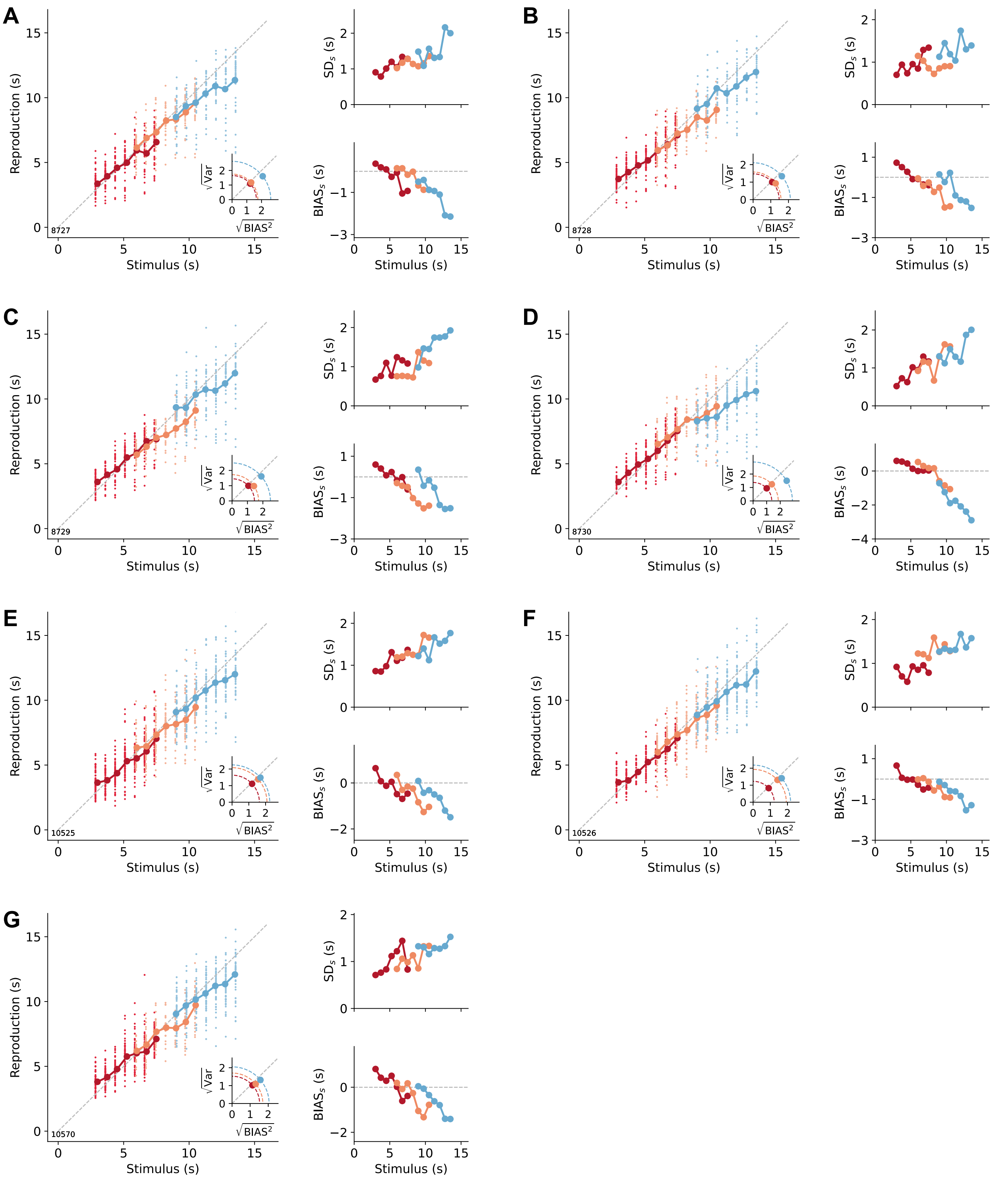

Figure S4. Time reproductions for all seven gerbils. (A-G) Each subfigure gives data for one animal. *Largest panels:* Reproduced values (small dots) and averages (large dots connected by a line) for each stimulus. Color identifies stimulus distribution as in the main text. Gray dashed lines mark identity line. *Inset:* Square roots of the average variance versus those for average squared bias for all three stimulus distributions. The distance from the origin of the coordinate system is the RMSE. Along the dashed quarter circles, RMSE is constant. *Upper right panels:* Standard deviation for each stimulus duration (SD_s). *Lower right panels:* Bias for each stimulus ($BIAS_s$). (E) replots the data of the example animal in Figure 1D-F.

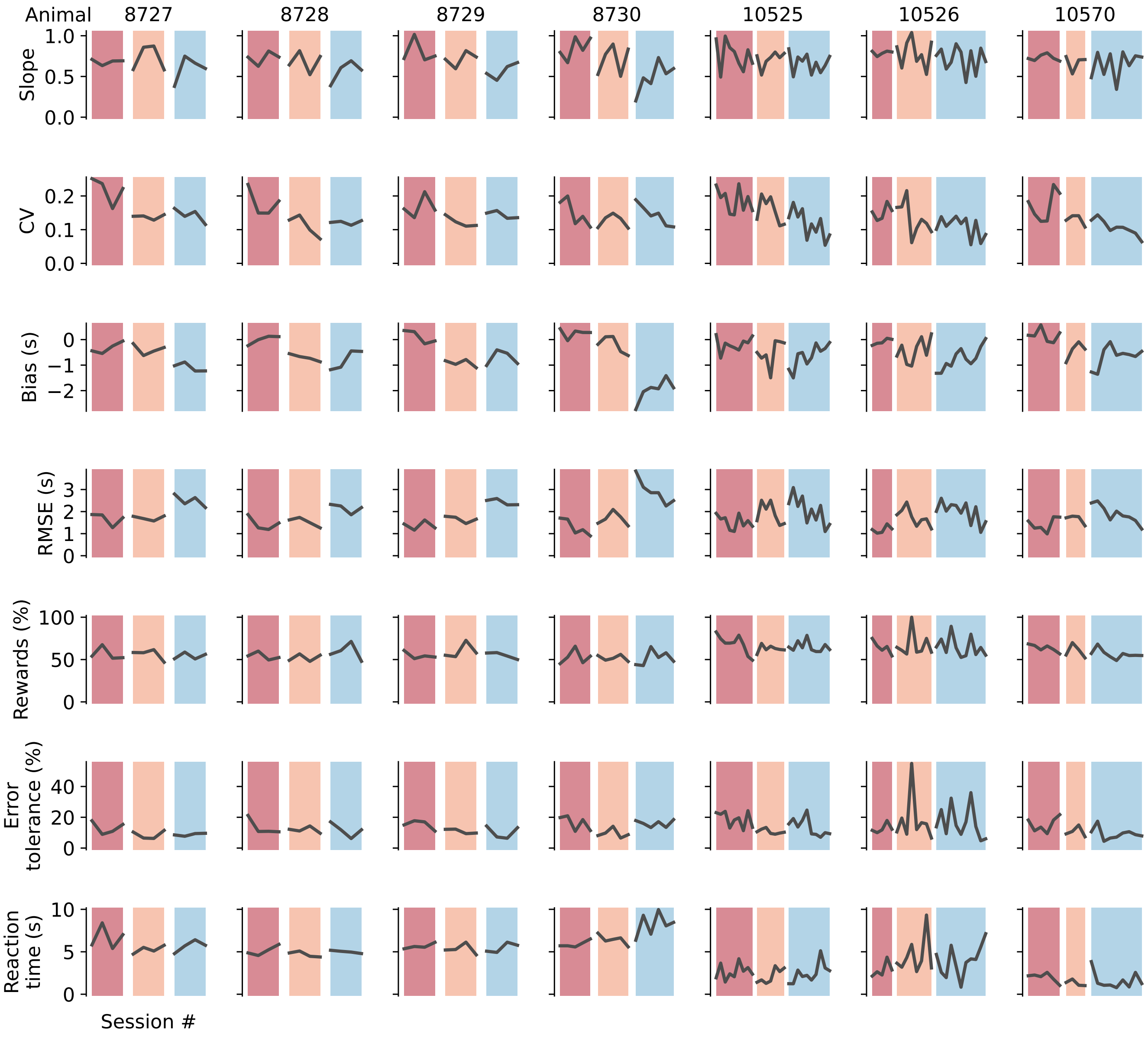

Figure S5. Session-wise analysis. Analysis parameters for each session and animal. Shading identifies short (red), intermediate (orange), and long (blue) range sessions. Sessions are in chronological order within each range. However, note that they did not occur one after the other for animals 10525, 10526 and 10570. Here, sessions from different ranges were interleaved.

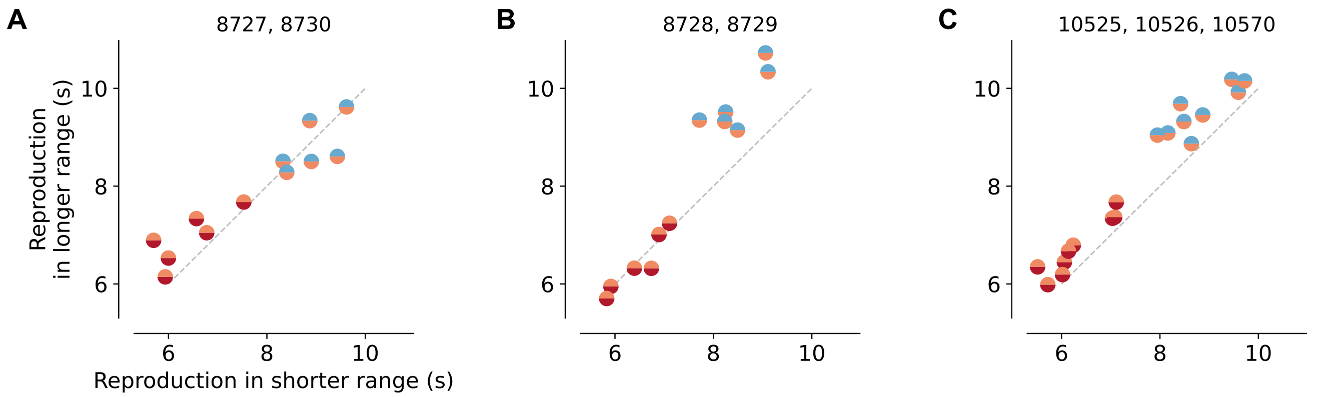

Figure S6. Reproductions for same stimuli embedded in different stimulus ranges grouped by session order. Reproduced values for stimuli that are part of the short and intermediate ranges (orange/red markers) or intermediate and long ranges (blue/orange markers). Gray dashed line marks equality line. All animals where first tested on the short range then animals in (A) performed the intermediate and finally the long range, those in (B) did long and then intermediate, and for the animals in (C) sessions in which intermediate and long stimulus ranges were presented were randomly interleaved.

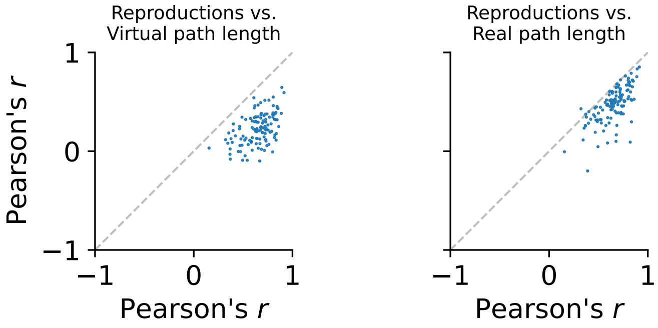

Figure S7. Time or distance reproduction? Pearson's correlations between stimulus and reproduction (abscissa) are compared to Pearson's correlations between stimulus and virtual, i.e. the distance covered in VR, or real path length, i.e. the distance covered on the treadmill (ordinate). Each dot represents a single session. Data was pooled across animals, stimulus ranges and sessions. Gray dashed line marks equality line. In both cases the correlation between stimulus and reproduction is larger, indicating that stimulus duration was reproduced and not mapped into a walking distance.
